# Human B cells and dendritic cells are susceptible and permissive to enterovirus D68 infection

**DOI:** 10.1128/msphere.00526-23

**Published:** 2024-01-23

**Authors:** Brigitta M. Laksono, Syriam Sooksawasdi Na Ayudhya, Muriel Aguilar-Bretones, Carmen W. E. Embregts, Gijsbert P. van Nierop, Debby van Riel

**Affiliations:** 1 Department of Viroscience, Erasmus MC, Rotterdam, the Netherlands; University of Zurich, Zurich, Switzerland

**Keywords:** enterovirus D68, enterovirus, viral pathogenesis, systemic spread, extra-respiratory infection, immune cells, B cells, dendritic cells

## Abstract

Enterovirus D68 (EV-D68) is predominantly associated with mild respiratory infections, but can also cause severe respiratory disease and extra-respiratory complications, including acute flaccid myelitis. Systemic dissemination of EV-D68 is crucial for the development of extra-respiratory diseases, but it is currently unclear how EV-D68 spreads systemically (viremia). We hypothesize that immune cells contribute to the systemic dissemination of EV-D68, as this is a mechanism commonly used by other enteroviruses. Therefore, we investigated the susceptibility and permissiveness of human primary immune cells for different EV-D68 isolates. In human peripheral blood mononuclear cells inoculated with EV-D68, only B cells were susceptible but virus replication was limited. However, in B cell-rich cultures, such as Epstein–Barr virus-transformed B-lymphoblastoid cell line (BLCL) and primary lentivirus-transduced B cells, which better represent lymphoid B cells, were productively infected. Subsequently, we showed that dendritic cells (DCs), particularly immature DCs, are susceptible and permissive for EV-D68 infection and that they can spread EV-D68 to autologous BLCL. Altogether, our findings suggest that immune cells, especially B cells and DCs, could play an important role in the pathogenesis of EV-D68 infection. Infection of these cells may contribute to systemic dissemination of EV-D68, which is an essential step toward the development of extra-respiratory complications.

**IMPORTANCE:**

Enterovirus D68 (EV-D68) is an emerging respiratory virus that has caused outbreaks worldwide since 2014. EV-D68 infects primarily respiratory epithelial cells resulting in mild respiratory diseases. However, EV-D68 infection is also associated with extra-respiratory complications, including polio-like paralysis. It is unclear how EV-D68 spreads systemically and infects other organs. We hypothesized that immune cells could play a role in the extra-respiratory spread of EV-D68. We showed that EV-D68 can infect and replicate in specific immune cells, that is, B cells and dendritic cells (DCs), and that virus could be transferred from DCs to B cells. Our data reveal a potential role of immune cells in the pathogenesis of EV-D68 infection. Intervention strategies that prevent EV-D68 infection of immune cells will therefore potentially prevent systemic spread of virus and thereby severe extra-respiratory complications.

## INTRODUCTION

Enterovirus D68 (EV-D68) is a small non-enveloped, positive single-stranded RNA virus that belongs to the family *Picornaviridae*, genus *Enterovirus*. Although EV-D68 infections induce predominantly mild upper respiratory tract symptoms, severe respiratory diseases were reported worldwide in 2014. In addition, in some individuals, EV-D68 was associated with neurological complications, of which acute flaccid myelitis (AFM) was reported most frequently (1–4). Since then, EV-D68 has caused biennial outbreaks of severe respiratory disease and AFM up to 2018 (5). EV-D68 circulation was limited during the pandemic, partly due to COVID-19 measures, but EV-D68-associated severe respiratory illnesses have been rising in several countries since 2021 and 2022 due to easing of COVID-19 measures (6, 7). Throughout the years, multiple clades of EV-D68 circulated, but so far there is little evidence for differences in the virulence of these viruses (1, 8, 9).

The ability of EV-D68 to disseminate from the respiratory tract, which is the primary replication site of the virus, to other organs, such as the central nervous system (CNS), is essential for the development of extra-respiratory complications. The virus may disseminate directly into the CNS via cranial nerves or to other organs via the hematogenous route. Retrograde axonal transport of EV-D68 has been shown in human induced pluripotent stem cell-derived motor neurons (10), suggesting that EV-D68 can use peripheral nerves that innervate the respiratory tract to spread to the CNS. Alternatively, the virus may spread via the circulation to other organ systems, before reaching the CNS. Studies in mice, ferrets, and patient samples have shown that the virus or viral RNA can be detected in the circulation (viremia and RNAemia, respectively) and extra-respiratory tissues (1, 11–17). In intranasally inoculated mice, virus and viral RNA were detected in the blood within 24 h post-inoculation (hpi), and in extra-respiratory tissues, such as the spleen and skeletal muscles (11, 12). In intranasally inoculated ferrets, virus was detected in axillary lymph nodes at multiple days post-inoculation (earliest detection at day five post-inoculation) (13). In humans, viral RNA has been detected in whole blood, plasma and serum of EV-D68 patients, but it is currently unknown how frequent viremia occurs during an EV-D68 infection (1, 14–20). However, despite the importance of viremia in the pathogenesis of EV-D68 infection, the mechanism that leads to viremia is poorly understood. In addition, it is unclear whether the virus detected in the circulation is a direct spill-over from the respiratory tract or whether virus first spreads to and replicates in other tissues, for example, lymphoid tissues, before disseminating into the circulation.

Other EVs can infect various immune cells with different replication efficiency. Coxsackievirus type B3 (CVB3) infects murine splenic B cells resulting in production of new virus particles (21). Poliovirus productively infects dendritic cells (DCs) and macrophages *in vitro* (human peripheral blood mononuclear cells [PBMC]) and *in vivo* (non-human primates) (22, 23). Echoviruses and EV-A71 have also been reported to infect human immature DCs (imDCs) and mature DCs (mDCs) (24, 25). Some EVs, for example, poliovirus and EV-A71 are known to replicate in lymphoid tissues, resulting in a sustained production of infectious viruses and subsequent spill-over into the circulation (26–30). For EV-D68, the role of immune cells and lymphoid tissues in the development of a viremia remains unclear. Previous studies have shown that EV-D68 productively infects several human immune cell lines, such as granulocytic (KG-1), monocytic (U-937), T (Jurkat and MOLT), and B (Raji) cell lines (31), suggesting that immune cells or lymphoid tissues may play a role in the development of EV-D68 viremia. Here, we investigated the susceptibility and permissiveness of human primary immune cells, B cells enriched models and monocytic-derived DCs to infection of EV-D68 from subclades A, B2, and A2 (previously known as D) isolated from respiratory samples of EV-D68-confirmed patients with respiratory disease in 2012 and 2014, to unravel the potential role of immune cells in the development of viremia and to investigate if there are clade-specific differences in the susceptibility and permissiveness of these cells. Subsequently, we investigated whether DCs can transmit virus to other immune cells, such as B cells.

## RESULTS

### Susceptibility and permissiveness of human immune cells to EV-D68 infections

**Fig 1:**
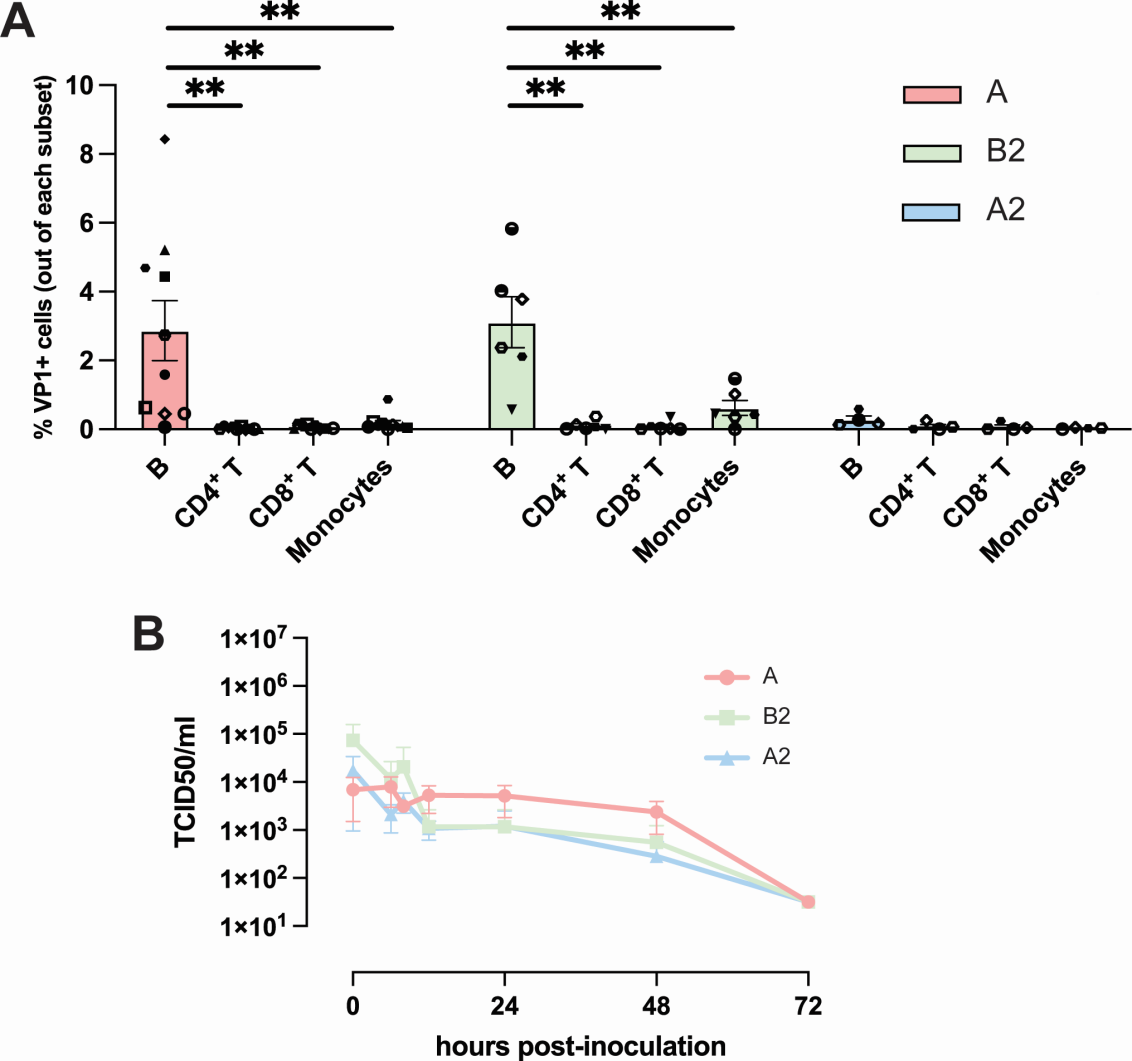
Susceptibility and permissiveness of human PBMC to EV-D68 infection. (**A**) Percentage of EV-D68 VP1^+^ immune cell subsets 24 h after inoculation with EV-D68 strains from subclades A (*n* = 10 donors), B2 (*n* = 6 donors), and A2 (*n* = 4 donors). Each symbol represents one donor. Statistical analysis was performed using unpaired *t*-test. Error bars denoted SEM. (**B**) Production of infectious virus in EV-D68-inoculated PBMC. PBMC, peripheral blood mononuclear cells; SEM, standard error of mean **P < 0.01.

To investigate whether human immune cell subsets are susceptible to EV-D68 infection, human PBMC were inoculated with EV-D68 strains from subclades A, B2, and A2 (previously known as D). At 24 hpi, we detected EV-D68/A- (mean 3% ± SEM 0.9) and EV-D68/B2- (3% ± 0.7) but not for EV-D68/A2-infected cells, based on intracellular expression of EV-D68 capsid protein VP1. In two donors, 1% of monocytes were susceptible to infection with EV-D68/B2. We did not observe any VP1^+^ cells in CD4^+^ nor CD8^+^ T cell population (Fig. 1A). Isotype controls were included, and to confirm that the VP1 signal that was detected was due to infection and not phagocytosis of the virus inoculum, heat-inactivated EV-D68/B2 was included as a control. We did not observe VP1^+^ cells in cells inoculated with heat-inactivated virus (Fig. S1).

Next, we investigated whether EV-D68 inoculation of PBMC resulted in the production of progeny viruses. Supernatant and cell lysate were collected and the presence of infectious virus particles at 0, 6, 8, 10, 24, 48, and 72 hpi was determined by virus titration. Despite the presence of infected cells after inoculation with EV-D68/A or EV-D68/B2, we did not observe an increase in infectious virus titer at any time point (Fig. 1B). As human PBMC consists only of 4%–14% of B cells (32), of which ~3% were infected by EV-D68, we cannot exclude the possibility that the replication in VP1^+^ B cells was below the detection limit of the assay.

**Fig 2:**
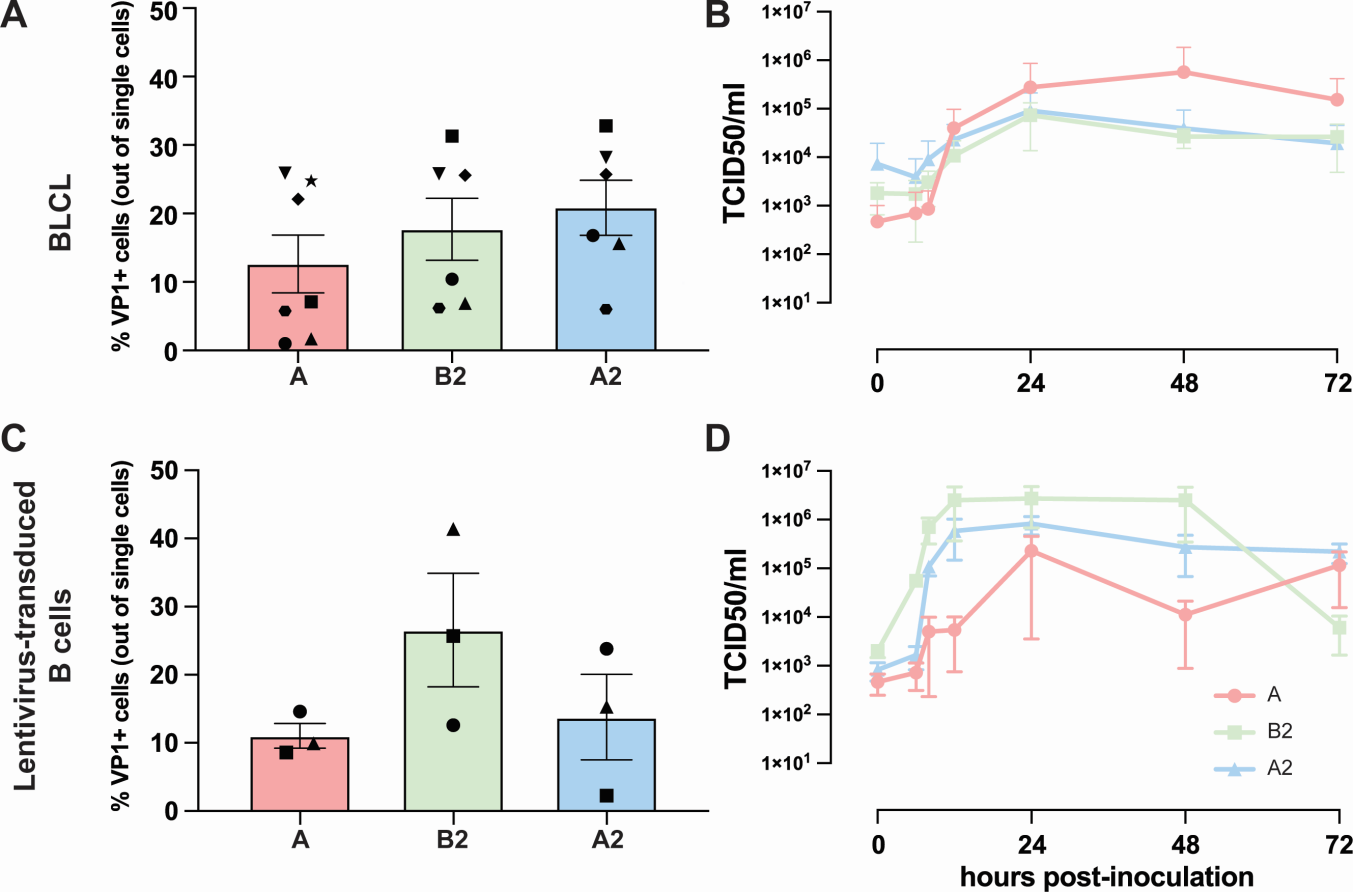
Susceptibility and permissiveness of B cell-rich models to EV-D68 infection. BLCL and lentivirus-transduced B cells were inoculated with EV-D68 strains from subclades A (*n* = 7), B2 (*n* = 6), and A2 (*n* = 6) as models for EV-D68 infection in B cell-rich environment. (**A**) Percentage of EV-D68 VP1^+^ cells at 24 hpi and (**B**) production of infectious viruses in EV-D68-inoculated BLCL over time, respectively. (**C**) Percentage of VP1^+^ cells at 24 hpi and (**D**) production of infectious viruses in EV-D68-inoculated lentivirus-transduced B-cells. Each symbol in (**A**) and (**C**) represents one donor. No statistically significant differences were observed in the percentages of VP1^+^ cells among the different virus subclades. Statistical analysis was performed using a one-way analysis of variance with multiple comparison test. Error bars denote SEM. BLCL, B-lymphoblastoid cell line; hpi, hours post-inoculation; SEM, standard error of mean.

In order to determine whether B cells are susceptible and permissive for EV-D68, we utilized two B cell-rich models. We first inoculated Epstein–Barr virus-transformed B-lymphoblastoid cell line (BLCL) with EV-D68 strains from subclades A, B2, and A2. The inoculation resulted in 13% ± 4 EV-D68/A, 18% ± 5 EV-D68/B2, and 21% ± 4 EV-D68/A2 VP1^+^ cells, without significant differences among the subclades. Furthermore, we observed variation among donors in the percentage of infected cells (Fig. 2A). Production of progeny viruses (~2–3 logarithmic increase of TCID_50_/mL within 24 h) was detected after inoculation with all three viruses, in which EV-D68/B2 and EV-D68/A2 replicated faster than EV-D68/A, albeit without any statistical differences (Fig. 2B). Heat-inactivated viruses were included as controls. We did not observe VP1^+^ cells in cells inoculated with heat-inactivated virus (Fig. S2).

To confirm the susceptibility and permissiveness of B cells, B cell clones were inoculated with EV-D68 strains from subclades A, B2, and A2. These cells originated from PBMC B cells and were lentivirus-transduced to express the germinal center-associated B cell lymphoma-6 (Bcl-6) and Bcl-xL in order to endow a stable proliferative state, thus representing germinal center B cells. This resulted in 11% ± 2 EV-D68/A, 27% ± 8 EV-D68/B2, and 14% ± 6 EV-D68/A2 VP1^+^ cells (Fig. 2C). The inoculation also resulted in production of new infectious viruses (~2–3 logarithmic increase within 10–24 h), without any statistical differences among the different EV-D68 subclades (Fig. 2D).

### Infection of BLCL is largely mediated by the presence of α2,3- and α2,6-linked SAs

**Fig 3:**
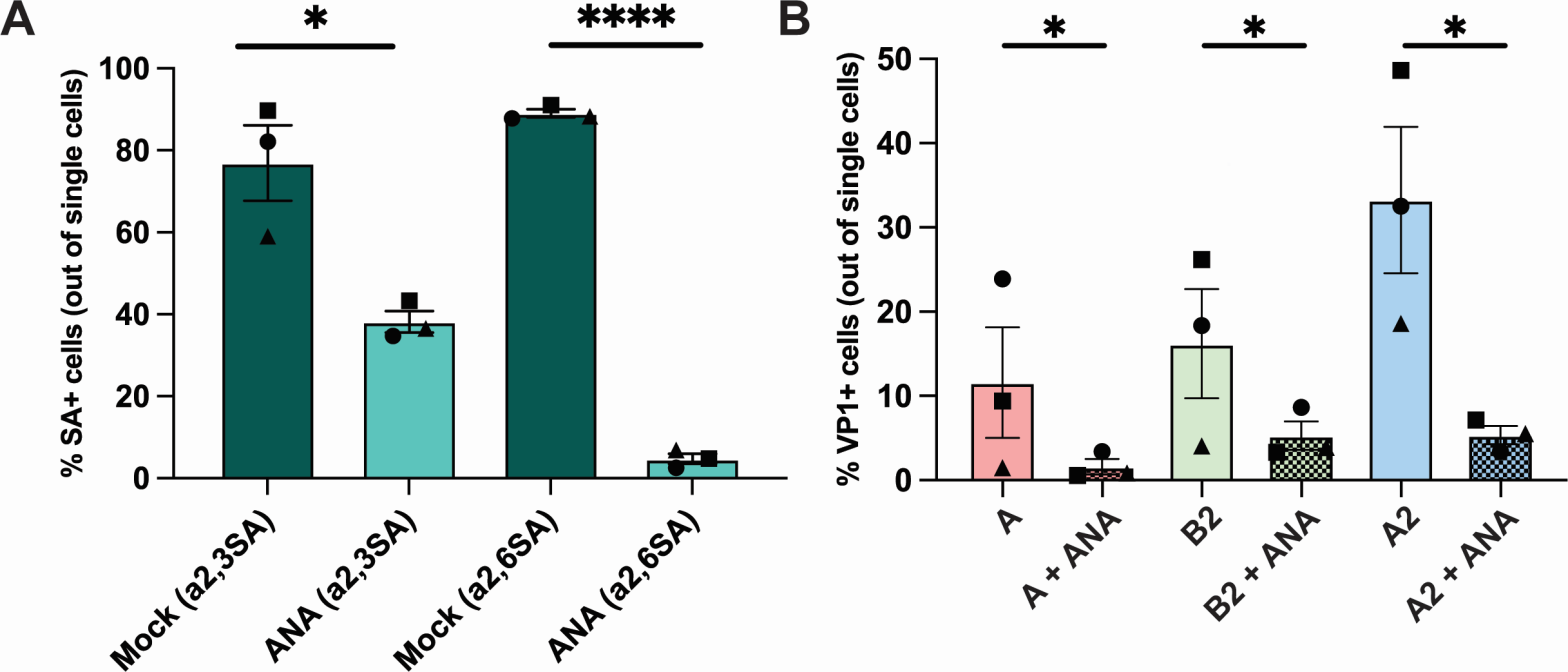
Percentages of α2,3-and α2,6-linked SAs^+^ and EV-D68 VP1^+^ BLCL upon neuraminidase treatment. (**A**) Percentage of BLCL expressed α2,3- (*n* = 3) and α2,6- (*n* = 3) linked SAs with and without ANA treatment. (**B**) Percentage of VP1^+^ BLCL (*n* = 3) with and without ANA treatment inoculated with EV-D68 from subclades A, B2, and A2, measured 24 hpi. Statistical analysis was performed with unpaired *t*-test. Error bars denote SEM. BLCL, B-lymphoblastoid cell line; ANA, *Arthrobacter ureafaciens* neuraminidase; SAs, sialic acids; hpi, hours post-inoculation; SEM, standard error of mean. *P < 0.05; ****P ≤ 0.0001.

Several immune cells, including B cells, express α2,6-linked and α2,3-linked sialic acids (SAs), which can function as receptors for EV-D68 to initiate binding and virus entry (33–35). To investigate whether α2,3- and α2,6-linked SAs mediate EV-D68 infection of BLCL, cell surface SAs were removed by *Arthrobacter ureafaciens* neuraminidase (ANA) prior to inoculation with EV-D68 strains. ANA hydrolyzes the terminal *N*- or *O*-acylneuraminic acids, which are α2,6- and α2,3-linked to oligo-, poly-, and mucopolysaccharides, glycoproteins and glycolipids, hence resulting in removal of SAs. Upon ANA treatment, the average percentages of α2,3-linked SA^+^ BLCL decreased from 77% ± 9 to 38% ± 3 and of α2,6-linked SA^+^ cells from 89% ± 1 to 5% ± 1 (Fig. 3A). ANA treatment of BLCL prior to inoculation resulted in a significant decrease of VP1^+^ cells at 24 hpi, with only 2% ± 1 EV-D68/A, 5% ± 2 EV-D68/B2, and 5% ± 1 EV-D68/A2 VP1^+^ cells (Fig. 3B).

### DCs are susceptible and permissive to EV-D68 infection

**Fig 4:**
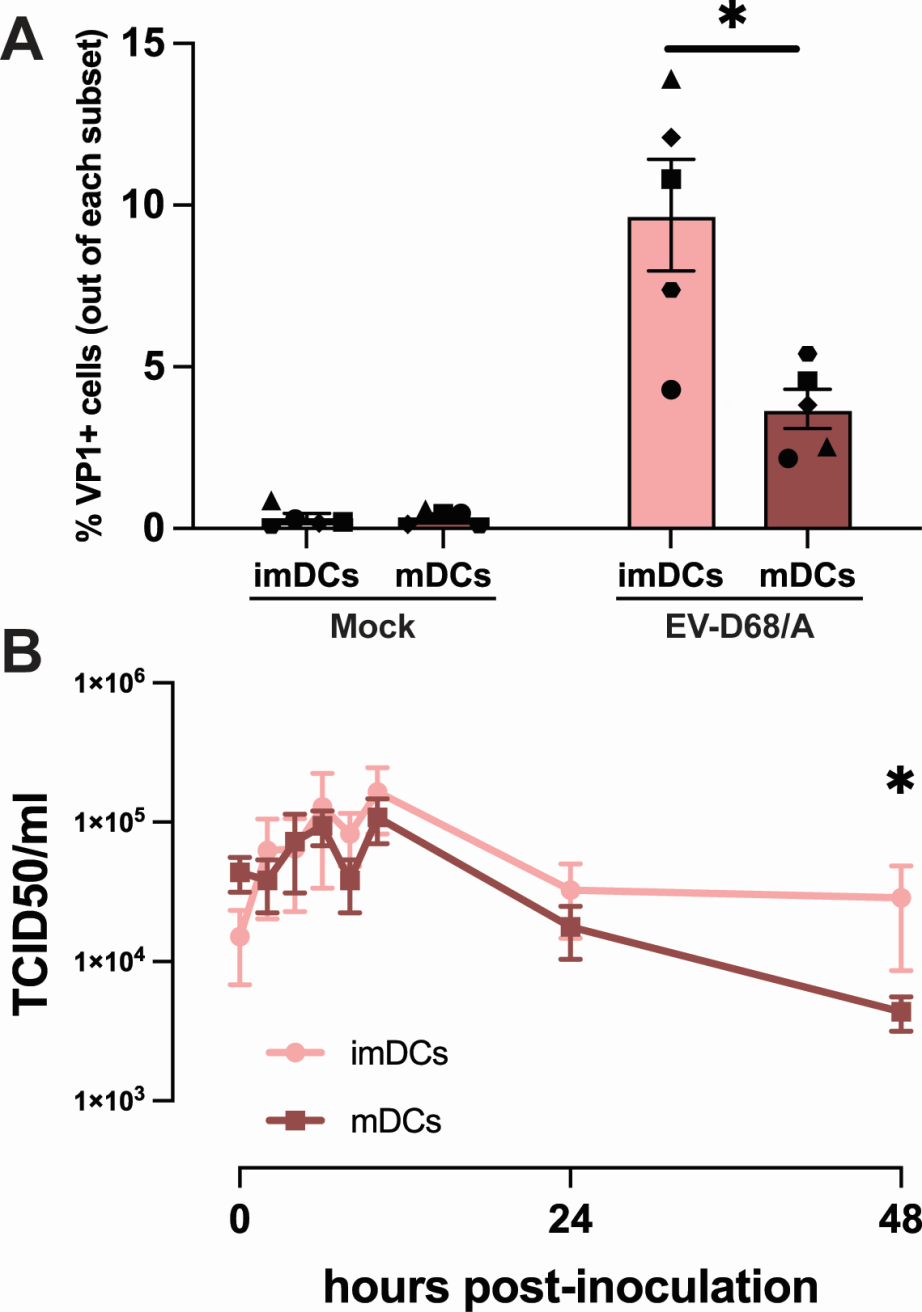
Susceptibility and permissiveness of imDCs and mDCs to EV-D68 infection. (**A**) Percentage of EV-D68 VP1^+^ imDCs (*n* = 5) and mDCs (*n* = 5) measured at 6 hpi. (**B**) Production of infectious viruses in EV-D68/A-inoculated imDCs (*n* = 3) and mDCs (*n* = 3). Samples for virus titration were collected at 0, 2, 4, 6, 8, 10, 24, and 48 hpi. All statistical analyses in this figure were performed with unpaired *t*-test. Error bars denote SEM. imDCs, immature dendritic cells; mDCs, mature dendritic cells; hpi, hours post-inoculation; SEM, standard error of mean. *P < 0.05.

DCs are a subset of immune cells that are attracted to sites of inflammation, but not abundantly present in PBMC (36). To investigate whether DCs are susceptible and permissive to EV-D68 infection, monocytes were differentiated in immature and mature DCs (imDCs and mDCs, respectively), and inoculated with EV-D68 strain from subclade A as a representative strain. Increased expression of maturation markers (HLA-DR, CD86, PD-L1, and CD83) was used to confirm differentiation of imDCs to mDCs (Fig. S3). The average percentage of VP1^+^ cells in imDCs (10% ± 1) was significantly higher than in mDCs (4% ± 1) at 6 hpi (Fig. 4A). From 0 to 2 hpi, virus titers seem to increase in the imDC, although this is not significant. This titer increase may also be caused by virus that initially attached to the cell surface, but was not internalized and released again into the supernatant. From 2 to 10 hpi, viral titers in the supernatants increased ~1 logarithmic TCID_50_/mL in imDCs and ~0.5 logarithmic TCID_50_/mL in mDCs inoculated with EV-D68/A, after which the virus titers decreased. Despite this decrease, at 48 hpi, virus titer in the supernatants of imDCs was significantly higher than in mDCs (Fig. 4B). Statistical analysis was performed for every time point, but only virus titers at 48 hpi showed a significant difference.

### imDCs can transfer EV-D68 to autologous BLCL

**Fig 5:**
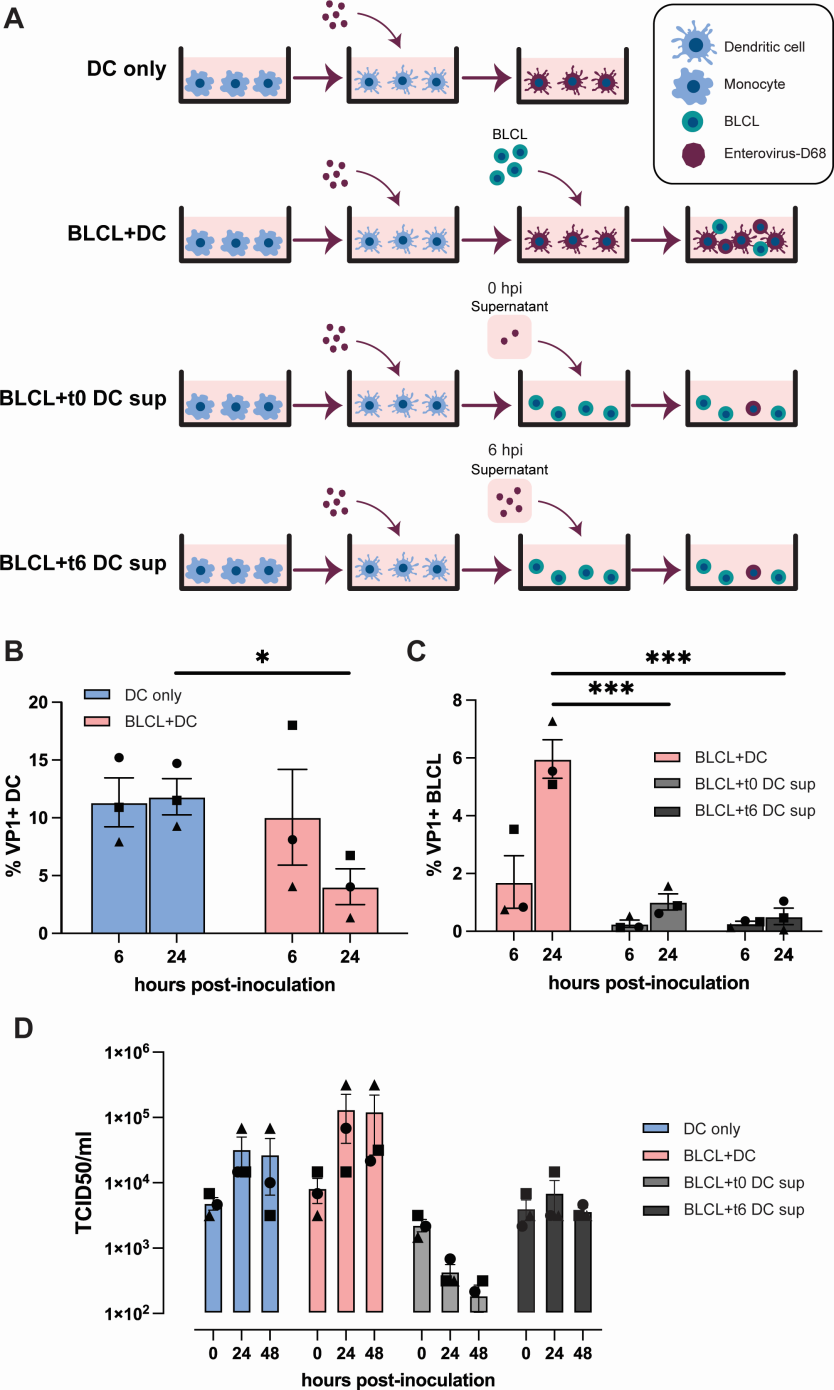
Co-culture of EV-D68-inoculated imDCs with their autologous BLCL. (**A**) Schematic representation of the experimental setup and controls. (**B**) Percentages of EV-D68 VP1^+^ imDCs and (**C**) BLCL in different culture conditions at 6 and 24 hpi. (**D**) Production of infectious viruses in different culture conditions. Statistical analysis in (**B**) was performed with unpaired *t*-test. Statistical analysis in (**C**) and (**D**) were performed with a one-way analysis of variance with multiple comparison test and compared to BLCL + t0 DC sup and BLCL + t6 sup, respectively. Error bars denote SEM. BLCL, B-lymphoblastoid cell line; imDCs, immature dendritic cells; hpi, hours post-inoculation; SEM, standard error of mean; BLCL + t0 DC sup, autologous BLCL co-cultured with supernatant collected from EV-D68/A-inoculated imDCs at 0 hpi. BLCL + t6 DC sup, autologous BLCL co-cultured with supernatant collected from EV-D68/A-inoculated imDCs at 6 hpi. *P < 0.05; ***P ≤ 0.001.

DCs are important for local antiviral responses and phagocytosis of viral antigens triggers DC migration to lymphoid tissues (36). Therefore, we investigated whether imDCs can transmit EV-D68 infection to B cells. EV-D68/A-inoculated imDCs were co-cultured with autologous BLCL (BLCL + DC), after which the percentages of VP1^+^ BLCLs were determined. The following controls were included: (1) EV-D68/A-inoculated imDCs (DC only) (2); BLCL cultured with supernatant from EV-D68/A-inoculated imDCs collected at 0 hpi, directly after the virus inoculation for 1 h and the subsequent washing steps (BLCL + t0 DC sup); and (3) BLCL cultured with supernatant collected around the peak of virus shedding (6 hpi) from EV-D68/A-inoculated imDCs (BLCL + t6 DC sup). The schematic representation of this experiment is presented in Fig. 5A. As observed previously, inoculation of imDCs in the absence of their autologous BLCL resulted in an average of 11% ± 2 VP1^+^ cells at 6 hpi and the percentage remained stable at 24 hpi. In imDCs co-cultured with BLCL, we observed an average percentage of 10% ± 4 VP1^+^ imDCs at 6 hpi, although this percentage decreased at 24 hpi (4% ± 2) (Fig. 5B). When BLCLs were inoculated with supernatants from EV-D68-inoculated imDCs collected at 0 or 6 hpi, only very few B cells were infected, with average percentages of 0.5% VP1^+^ BLCLs in BLCL + t0 DC sup and 1% VP1^+^ BLCLs in BLCL + t6 DC sup at 24 hpi. When BLCLs were co-cultured with EV-D68-inoculated imDCs, the percentage of infected BLCL increased from 2% ± 1 at 6 hpi to 6% ± 1 at 24 hpi (Fig. 5C).

The inoculation of imDCs in the absence of BLCL resulted in production of new infectious virus particles (~1 logarithmic increase of TCID_50_/mL) within 24 h. Detection of infectious virus particles in the supernatants of the BLCL + DC co-culture showed a stably higher virus titer (~1–1.5 logarithmic increase of TCID_50_/mL) within the same period of time. Very few, if any, new infectious virus particles were produced by BLCL inoculated with t0 or t6 DC supernatants (Fig. 5D). Statistical analysis was performed but there was no significant difference in virus titer at 0, 24, and 48 hpi. Together, the results suggested that imDCs might be capable to transmit virus to other susceptible immune cells, such as B cells, which subsequently become productively infected.

## DISCUSSION

The systemic dissemination, either cell-free or cell-associated, of EV-D68 is an essential step for extra-respiratory spread of the virus and the development of associated complications, such as AFM. In this study, we reveal the potential role of immune cells in the pathogenesis of EV-D68 infection. We show that human B cells and DCs are susceptible and permissive to EV-D68 infection and that DCs may play a role in the transmission of virus to B cells.

Immune cells are susceptible to infection of other members of *Picornaviridae*. Coxsackievirus type B infects murine splenic B cells resulting in production of new virus particles (21), and poliovirus productively infects DCs and macrophages *in vitro* (human PBMC) and *in vivo* (non-human primates) (22, 23). Echoviruses and EV-A71 have also been reported to infect human imDCs and mDCs (24, 25). Here, we showed that human B cells and DCs are susceptible and permissive to EV-D68 infection, which fits with findings in a cohort study that detected enterovirus RNA in peripheral blood B cells and DCs (37). In B cells, this tropism is facilitated by the presence of α2,6- and/or α2,3-linked SAs. Productive infection was only detected in more activated B cell populations, that is, BLCLs and lentivirus-transduced B cells, of which the latter resembles activated germinal center B cells (38). The detection of VP1 and the absence of new infectious virus particles in PBMC B cells can be explained by several potential mechanisms: (1) B cells are present in low number in the circulation (~4%–14%) (32). If only ~1%–8% of PBMC B cells become infected, viral replication will potentially occur below the detection limit of our titration assay (2); EV-D68 fails to replicate in PBMC B cells. The precise mechanisms that lead to this failure (e.g., failure in viral uncoating, formation of replication organelle, or virion assembly) are currently unknown. In addition, we observed that imDCs were more susceptible and permissive to EV-D68 infection than mDCs. It is possible that the different susceptibility to EV-D68 infection between imDCs and mDCs is due to the higher expression of α2,6-linked SA on imDCs than mDCs (39). It is also likely that the activation or maturation state of immune cells plays an important role in the susceptibility for EV-D68 infection. This has been shown for other enteroviruses, where activated and/or immortalized B cells were more susceptible to CVB3 infection than primary immune cells, and imDCs were more susceptible to poliovirus infection than mDCs (22, 40). As for the permissiveness to EV-D68 infection, or enteroviruses in general, metabolic activity of different immune cell subtypes may play a role and therefore requires further investigation.

We observed differences in the susceptibility and permissiveness to EV-D68 infection among viruses or donors included in this study. Differences in the susceptibility to different EV-D68 subclades were observed in B cells within the PBMCs, but this was not observed in BLCLs and lentivirus-transduced B cells. Donor variation was observed in PBMCs and all B cell models, and in two PBMC donors, we observed a low percentage of EV-D68/B2-infected monocytes. The underlying mechanism for these differences among donors and possibly among different virus subclades, as well as their association with the risk of the development of extra-respiratory diseases, are still unclear.

How EV-D68 spreads from the respiratory tract to the CNS is still poorly understood. Two potential routes can be utilized by the virus: first, through direct infection of peripheral nerves that innervate the respiratory tract, oral cavity, or tongue, and subsequent retrograde axonal transport of the virus to the CNS; second, through viremia. Once the virus enters the circulation, it can spread and replicate in other organs, such as skeletal muscles. In muscle cells, virus could enter motor neurons via the neuromuscular junction and subsequently spread to the CNS via retrograde axonal transport. Alternatively, the virus spreads from the circulation into the CNS through the blood-brain-barrier or blood-cerebrospinal fluid barrier. Whether EV-D68 utilizes one dissemination route or more to invade the CNS needs to be investigated further.

Virus replication within immune cells and/or lymphoid tissues is likely important for the development of a viremia. Due to the susceptibility and permissiveness of immune cells to EV-D68 infection, an immune cell-rich environment, such as a lymphoid tissue, can serve as a secondary replication site for EV-D68, from where the virus can spread into the circulation. Moreover, our data suggests that EV-D68 can be transferred from DCs to B cells via cell-to-cell spread. Lymphoid tissues are densely populated by DCs and B cells, which facilitated interactions between these cells, making the tissues an ideal site for EV-D68 replication. Since poliovirus viremia is essential for virus spread to the CNS and the subsequent development of poliomyelitis (41), it can be speculated that EV-D68 viremia is one of the mechanisms that is essential for virus entry into the CNS and the subsequent development of AFM (3, 14). In addition, a viremia could lead to virus spread to other organ system contributing to non-neurological complications associated with EV-D68 infection, including acute gastroenteritis, myocarditis, and skin rash (42–44). Prevention of spread to, or productive infection of lymphoid tissues may prevent systemic dissemination, as observed in poliovirus infection, in which vaccination prevents viral spread to other organs (45).

**Fig 6:**
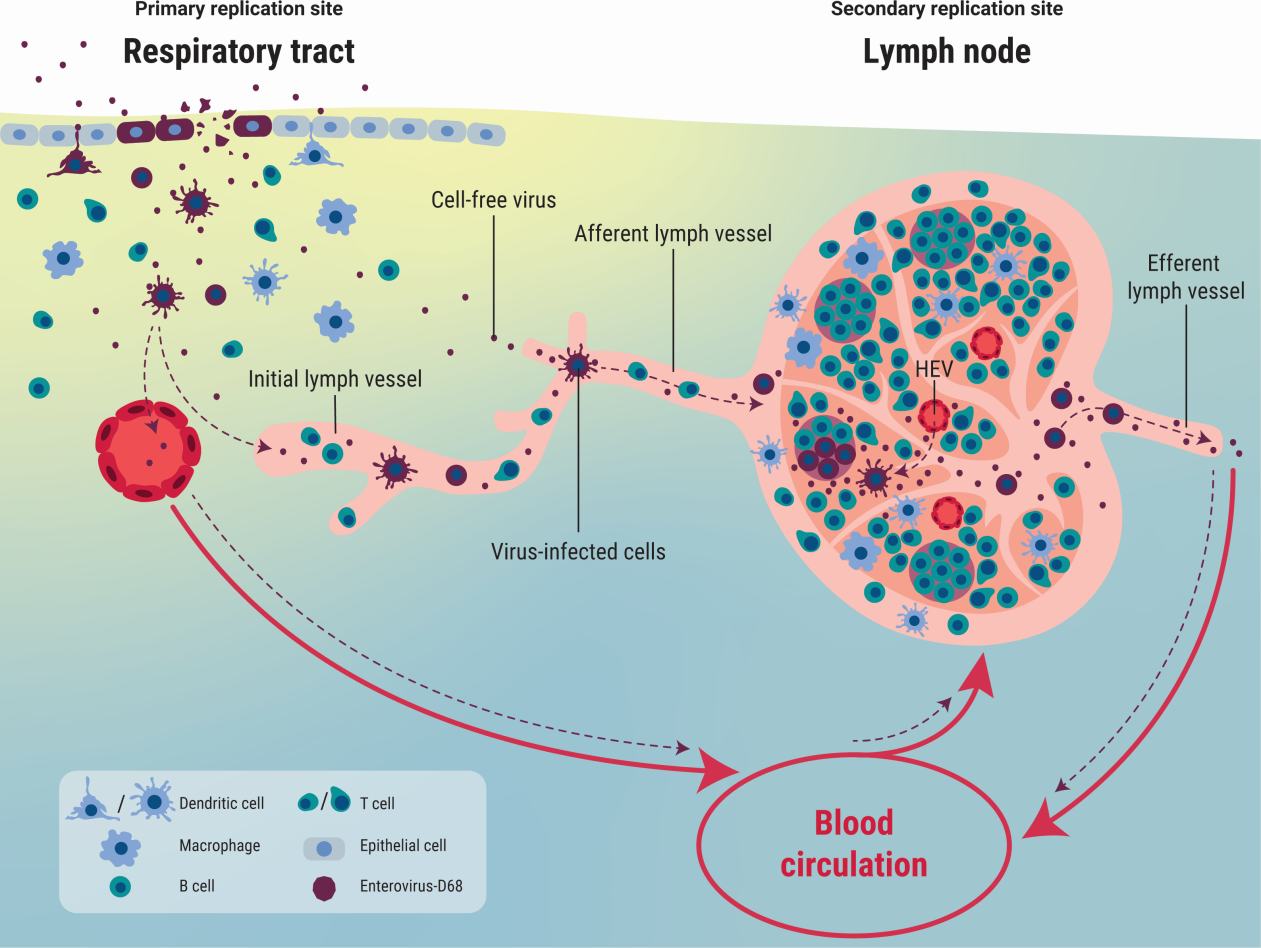
Proposed model for systemic dissemination of EV-D68. EV-D68 enters the respiratory tract and initially infects respiratory epithelial cells. The infection will result in the recruitment of immune cells, including imDCs. Subsequently, cell-free EV-D68 can spread to lymphoid tissues by spilling over into the circulatory or lymphatic system. Alternatively, it can infect imDCs, which can transfer the virus to lymphoid tissues. These lymphoid tissues can function as a secondary replication site, where EV-D68 infects mDCs and B cells. From this site, cell-free virus or virus-infected immune cells can enter the blood circulation and spread virus to other tissues. HEV, high endothelial venule; red arrow, blood circulation; purple dotted arrow, potential routes of EV-D68 spread.

Based on our findings, we propose a model that explains the systemic dissemination of EV-D68 (Fig. 6). EV-D68 infects respiratory epithelial cells, which results in the production of infectious virus particles and the recruitment of immune cells, such as imDCs. From the respiratory tract, cell-free virus can spill over into the circulatory and lymphatic systems and spread into lymphoid tissues. Alternatively, imDCs can become infected, and migrate to the lymphoid tissues, where they release newly produced viruses or spread virus to resident DCs or B cells. Lymphoid tissues can be the secondary replication site for EV-D68, from where the virus is released in the bloodstream. Prevention of virus spread to and amplification in lymphoid tissues can therefore prevent the development of a subsequent viremia and severe extra-respiratory complications caused by EV-D68.

## MATERIALS AND METHODS

### Cells

Human PBMC were isolated from blood (*n* = 10 healthy donors) by Ficoll density gradient centrifugation. BLCL was established from seven donors by transformation with Epstein–Barr virus as previously described (46). PBMC and BLCL were cultured in RPMI-1640 medium (Capricorn) supplemented with 10% fetal bovine serum (FBS) and 100 IU/mL of penicillin, 100 µg/mL of streptomycin, and 2 mM L-glutamine (PSG). After virus inoculation, supernatants and cell lysates were collected and frozen and thawed three times prior to sample processing.

Germinal center-like B cell clones were generated from three donors. Synthetic cDNA encoding a self-cleaving polyprotein Bcl-6.t2A.Blc-xL to express the germinal center B cell-associated transcription factors Bcl-6 and Bcl-xL was synthesized (B6L; Integrated DNA Technologies) and cloned in pENTR/D-TOPO (Thermo Fisher), generating pENTR.B6L (38, 47). The B6L cDNA was subsequently transferred to pLenti6.3/V5-DEST using Gateway LR Clonase II (Thermo Fisher), generating pLV-B6L. Subsequently, lentiviral vector stocks (LV-B6L) were generated, all according to manufacturer's instructions (Thermo Fisher). Primary CD19^+^ B cells were isolated from human PBMC using the EasySep human CD19 positive selection kit II (StemCell Technologies) and transduced using LV.B6L as described elsewhere (38). LV-B6L transduced B cells were cultured in AIM-V AlbuMAX medium (Gibco) supplemented with 10% FBS, PSG, 50 µM beta-mercaptoethanol (Sigma), 25 ng/mL recombinant human IL-21 (Peprotech), and growth-arrested 40 gray gamma-irradiated L-CD40L feeder cells. Every 3–4 days, new culture medium was added with 25 ng/mL recombinant human IL-21 and new growth-arrested 40 gray gamma-irradiated L-CD40L feeder cells. Clonal B cell cultures from each donor were generated using limiting dilution. Absence of antibody reactivity toward EV-D68 was confirmed to exclude antibody-mediated effects on EV-D68 infection (data not shown). After virus inoculation, supernatants and cell lysates were collected and frozen and thawed three times prior to sample processing.

Human rhabdomyosarcoma (RD) cells were cultured in DMEM (Lonza) supplemented with 10% FBS and PSG.

### Viruses

EV-D68 strains included in this study were isolated from clinical specimens at the National Institute of Public Health and the Environment (RIVM), Bilthoven, the Netherlands. The viruses were isolated on RD cells (ATCC) at 33°C at RIVM from respiratory samples from patients with EV-D68-associated respiratory disease. Virus stocks for the studies were grown in RD cells at 33°C in 5% CO_2_. The viruses included in this study with virus reference number, year of isolation an accession number are as follows: subclade A (or A1; 4311200821; 2012, accession number MN954536), subclade A2 (previously known as D; 4311400720; 2014, accession number MN954537), and subclade B2 (4311201039; 2012, accession number MN954539). All virus stocks were sequenced for their whole genome, and there was no evidence for cell culture adaptive mutations. For heat-inactivated virus controls, the same viruses from subclades A, B2, or A2 were heat-inactivated at 70°C for 30 min prior to inoculation.

### Virus titration

Virus titers were assessed by end-point titrations in RD cells and were expressed in median tissue culture infectious dose (TCID_50_/mL). In brief, 10-fold serial dilutions of a virus stock were prepared in triplicate and inoculated onto a monolayer of RD cells. The inoculated plates were incubated at 33°C in 5% CO_2_. Cytopathic effect was determined at day 5, and virus titers were determined using the Spearman–Kärber method (48).

### Differentiation of monocyte-derived DCs

Human monocytes were isolated from buffy coats (*n* = 5 donors) by Ficoll density gradient centrifugation and selected for CD14^+^ cells using magnetic beads. Monocytes were cultured in RPMI-1640 medium supplemented with 1× Glutamax*,* 10% FBS, 100 IU/mL of penicillin, and 100 µg/mL of streptomycin. Subsequently, monocytes were differentiated into imDCs in the presence of human interleukin 4 (IL-4; 20 ng/mL; PEPROTECH; 200-04) and human granulocyte-macrophage colony-stimulating factor (GM-CSF; 20 ng/mL; MILTENYI BIOTEC; 130-093-866). At day 5, monocyte-derived imDCs were further differentiated into mature DCs by adding lipopolysaccharide (LPS; 1 µg/mL; Thermo Fisher Scientific; L8274-10MG) into the medium. mDCs were defined by the increased expression of HLA-DR, CD86, PD-L1, and CD83 compared to imDCs (Fig. S3) and the determination of the cellular marker expression by flow cytometry is described below.

### EV-D68 infection of PBMC, BLCL, and lentivirus-transduced B cells

Freshly isolated PBMCs, BLCLs, or lentivirus-transduced B cells (1 × 10^6^ cells) were inoculated with EV-D68 or heat-inactivated EV-D68 at multiplicity of infection (MOI) of 0.1 for 1 h at 37°C in 5% CO_2_. The inoculum was removed and cells were seeded in new medium into a U-bottomed 96-well plate. Cells and supernatants were collected at 0, 6, 8, 12, 24, 48, and 72 hpi. The collected specimens were frozen and thawed three times to allow release of intracellular virus before further used for virus titration. Cells were also collected at 6, 24, 48, and 72 hpi for detection of intracellular capsid protein VP1 (10  µg/mL; GeneTex; GTX132313) by flow cytometry as described below. Normal rabbit serum was included as VP1 isotype control in PBMC.

### Removal of cell surface SAs on BLCL

BLCLs were incubated with 50 mU/mL *A. ureafaciens* neuraminidase (Roche) in serum-free medium for 2 h at 37°C in 5% CO_2_. Removal of α (2,3)-linked and α (2,6)-linked SAs on the cell surface was verified by staining with biotinylated *Maackia amurensis* lectin (MAL) I (5  µg/mL; Vector Laboratories; B-1265-1) or fluorescein-labeled *Sambucus nigra* lectin (SNA) (5  µg/mL; EY Laboratories; BA-6802-1), respectively. Biotin was detected using a streptavidin-conjugated AlexaFluor488 (5  µg/mL; Thermo Fisher Scientific; S11223). Virus and mock inoculations in non-enzymatic-treated cells were included as positive and negative infection controls, respectively. Infection of BLCL in different conditions was performed as describe above and intracellular capsid protein VP1 were detected by flow cytometry as described below.

### EV-D68 inoculation of DCs and co-culture of DCs and BLCLs

ImDCs and mDCs in a flat-bottomed 96-well plate (1 × 10^5^ cells/well) were inoculated with EV-D68/A at MOI of 1. After 1 h, the inoculum was removed and the monocytes-derived imDCs were supplemented with complete RPMI-1640 containing human IL-4 and GM-CSF, while mDCs were supplemented with complete RPMI-1640 containing human IL-4, GM-CSF, and LPS. Cells and supernatants were collected at 0, 2, 4, 6, 8, 10, 24, and 48 hpi for virus titration or at 6 hpi for detection of intracellular expression of capsid protein VP1 by flow cytometry as described below.

For co-culture assay, infected imDCs (1 × 10^5^ cells/well) as describe above were incubated with (2 × 10^5^ BLCL/well; BLCL + DC) or without autologous BLCL (DC only). To investigate whether imDCs transfer virus particles directly to autologous BLCL or indirectly by release of infectious virus, the supernatant from imDCs that were infected with EV-D68/A for 6 h, were transferred to autologous BLCL (BLCL + t6 DC sup). As a control, to show that infected BLCL is not due to the leftover of virus inoculum, supernatant from the last washing step in infected imDCs was transferred to the autologous BLCL (BLCL + t0 DC sup). The schematic representation of the co-culture assay is presented in Fig. 5A. The intracellular expression of capsid protein VP1 was detected at 6 and 24 hpi by flow cytometry as described below. Cells and supernatant were collected to detect infectious virus particles at different time points.

### Flow cytometry

**Fig 7:**
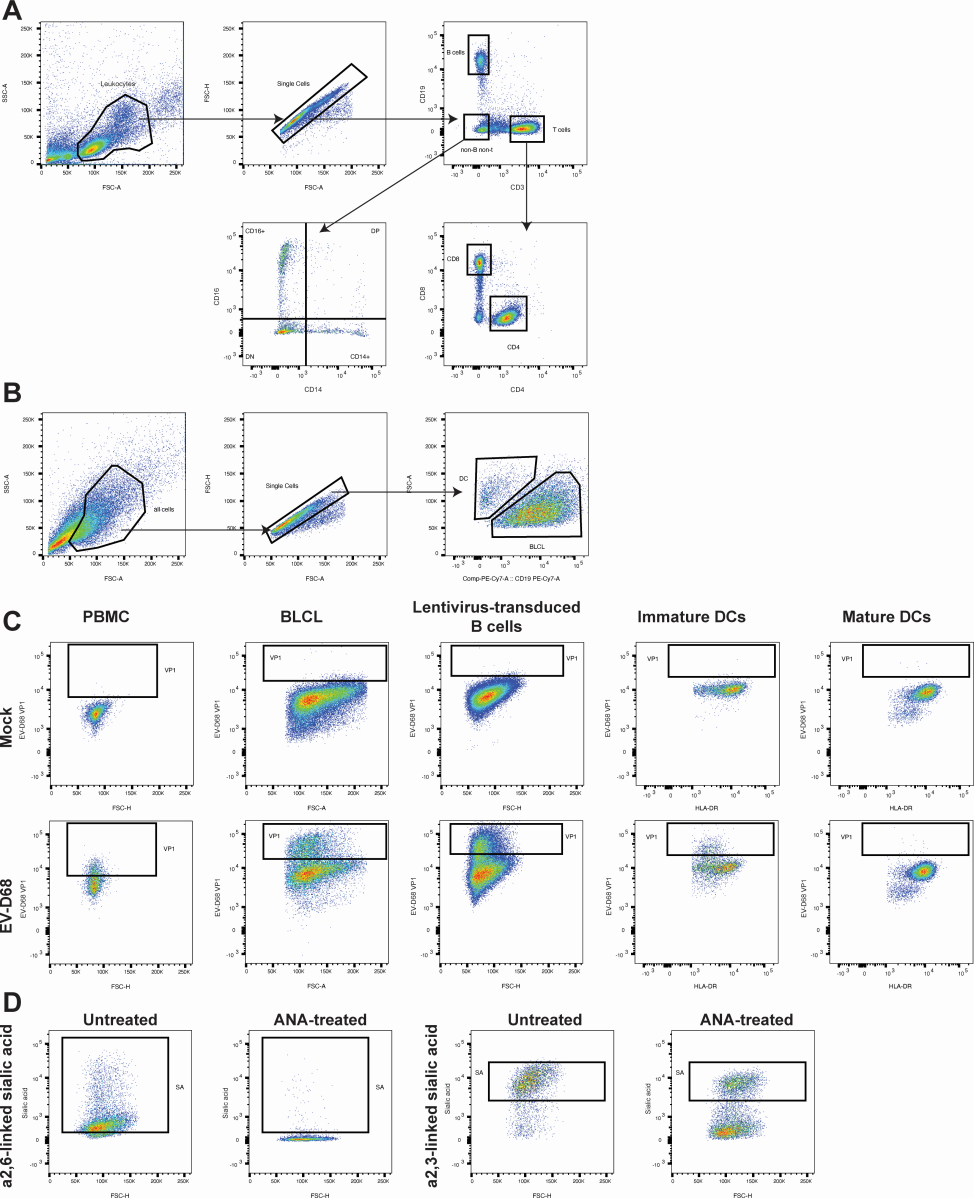
Representative gating strategies for flow cytometry analyses. (**A**) Gating strategy to determine peripheral blood mononuclear cell subpopulations. B cells were defined as CD3^−^CD19^+^ cells; CD4^+^T cells as CD3^+^CD19^−^CD4^+^CD8^−^ cells; CD8^+^T cells as CD3^+^CD19^−^CD4^−^CD8^+^ cells; and monocytes as CD3^−^CD19^−^CD14^−/+^CD16^−/+^ cells. (**B**) Gating strategy of EV-D68-inoculated immature dendritic cells (imDCs) co-cultured with autologous B-lymphoblastoid cell line (BLCL). imDCs were defined as CD19^−^ cells; BLCL were defined as CD19^+^ cells. (**C**) Gating strategy to determine EV-D68 VP1^+^ cells. PBMC, peripheral blood mononuclear cells; BLCL, B-lymphoblastoid cell line; DCs, dendritic cells. (**D**) Gating strategy to determine percentages of α2,3- and α2,6-linked sialic acid^+^ (SAs^+^) BLCL. Percentages of SA^+^ cells were determined at 0 h post-inoculation. ANA, *Arthrobacter ureafaciens* neuraminidase; BLCL, B-lymphoblastoid cell line.

For determination of immune cell phenotypes, human PBMC were incubated with monoclonal antibodies against CD19 (PE-Cy7; Beckman Coulter; IM3628), CD3 (PerCP; BD Biosciences; 345766), CD4 (V450; BD Biosciences; 560811), CD8 (AmCyan; BD Biosciences; 339188), CD14 (BV711; BD Biosciences; 740773), and CD16 (AlexaFluor647; BD Biosciences; 302020). For determination of DC phenotypes, DCs were incubated with monoclonal antibodies against HLA-DR (Pacific Blue; BioLegend; 307624), CD83(PE-Cy7; BioLegend; 305326), CD86 (AF647; BioLegend; 305416), and PD-L1 (BV785; BioLegend; 320736). For determination of cell phenotypes in DC-BLCL co-cultures, the cells were incubated with monoclonal antibodies against HLA-DR (Pacific Blue; BioLegend; 307624), CD86 (AF647; BioLegend; 305416), and CD19 (PE-Cy7; Beckman Coulter; IM3628). Cells were fixed and permeabilized with BD Cytofix/Cytoperm Fixation and Permeabilization kit according to the manufacturer's instructions (BD Biosciences). The presence of intracellular capsid protein VP1 was determined by staining with polyclonal rabbit anti-EV-D68 VP1 (10 µg/mL; Genetex; GTX132313) and goat anti-rabbit IgG (FITC; BD Biosciences; 554020). Flow cytometry was performed using BD FACS Lyric (BD Biosciences, USA). Data were acquired with BD Suite software and analyzed with FlowJo software. Gating strategies to define different cell phenotypes and to define VP1^+^ cells are presented in Fig. 7.

### Statistical analyses

Statistical analyses were performed using GraphPad Prism 9.0 software (La Jolla, CA, USA). Specific tests are described in the figure legends. *P* values of  ≤ 0.05 were considered significant. All data were expressed as SEM.

## Data Availability

The viruses included in this study are available under accession numbers MN954536, MN954537, and MN954539.

## SUPPLEMENTAL MATERIAL

Fig. S1 msphere.00526-23-s0001.tif PBMC VP1 staining controls.

Fig. S2 msphere.00526-23-s0002.tif BLCL heat-inactivated controls.

Fig. S3 msphere.00526-23-s0003.tif Maturation markers of immature and mature DCs.

Supplemental Material Legends msphere.00526-23-s0004.docx Legends for Fig. S1-S3.

ASM does not own the copyrights to Supplemental Material that may be linked to, or accessed through, an article. The authors have granted ASM a non-exclusive, world-wide license to publish the Supplemental Material files. Please contact the corresponding author directly for reuse.

## References

[1] Greninger AL, Naccache SN, Messacar K, Clayton A, Yu G, Somasekar S, Federman S, Stryke D, Anderson C, Yagi S, Messenger S, Wadford D, Xia D, Watt JP, Van Haren K, Dominguez SR, Glaser C, Aldrovandi G, Chiu CY. 2015. A novel outbreak enterovirus D68 strain associated with acute flaccid myelitis cases in the USA (2012-14): a retrospective cohort study. Lancet Infect Dis 15:671–682. doi:10.1016/S1473-3099(15)70093-9 25837569 PMC6027625

[2] Messacar K, Abzug MJ, Dominguez SR. 2016. The emergence of enterovirus-D68. Microbiol Spectr 4. doi:10.1128/microbiolspec.EI10-0018-2016 27337448

[3] Messacar K, Schreiner TL, Van Haren K, Yang M, Glaser CA, Tyler KL, Dominguez SR. 2016. Acute flaccid myelitis: a clinical review of US cases 2012-2015. Ann Neurol 80:326–338. doi:10.1002/ana.24730 27422805 PMC5098271

[4] Messacar K, Pretty K, Reno S, Dominguez SR. 2019. Continued biennial circulation of enterovirus D68 in Colorado. J Clin Virol 113:24–26. doi:10.1016/j.jcv.2019.01.008 30825833

[5] Kramer R, Sabatier M, Wirth T, Pichon M, Lina B, Schuffenecker I, Josset L. 2018. Molecular diversity and biennial circulation of enterovirus D68: a systematic screening study in Lyon, France, 2010 to 2016. Euro Surveill 23:1700711. doi:10.2807/1560-7917.ES.2018.23.37.1700711 30229724 PMC6144471

[6] Benschop KS, Albert J, Anton A, Andrés C, Aranzamendi M, Armannsdóttir B, Bailly J-L, Baldanti F, Baldvinsdóttir GE, Beard S, et al.. 2021. Re-emergence of enterovirus D68 in Europe after easing the COVID-19 lockdown, September 2021. Euro Surveill 26:45. doi:10.2807/1560-7917.ES.2021.26.45.2100998 PMC8646978 34763750

[7] Ma KC, Winn A, Moline HL, Scobie HM, Midgley CM, Kirking HL, Adjemian J, Hartnett KP, Johns D, Jones JM, Lopez A, Lu X, Perez A, Perrine CG, Rzucidlo AE, McMorrow ML, Silk BJ, Stein Z, Vega E, Hall AJ, New Vaccine Surveillance Network Collaborators. 2022. Increase in acute respiratory illnesses among children and adolescents associated with rhinoviruses and enteroviruses, including enterovirus D68 - United States, July-September 2022. MMWR Morb Mortal Wkly Rep 71:1265–1270. doi:10.15585/mmwr.mm7140e1 36201400 PMC9541033

[8] Wang G, Zhuge J, Huang W, Nolan SM, Gilrane VL, Yin C, Dimitrova N, Fallon JT. 2017. Enterovirus D68 subclade B3 strain circulating and causing an outbreak in the United States in 2016. Sci Rep 7:1242. doi:10.1038/s41598-017-01349-4 28455514 PMC5430842

[9] Yip CCY, Lo JYC, Sridhar S, Lung DC, Luk S, Chan K-H, Chan JFW, Cheng VCC, Woo PCY, Yuen K-Y, Lau SKP. 2017. First report of a fatal case associated with EV-D68 infection in Hong Kong and emergence of an interclade recombinant in China revealed by genome analysis. Int J Mol Sci 18:18. doi:10.3390/ijms18051065 PMC5454976 28509856

[10] Hixon AM, Clarke P, Tyler KL. 2019. Contemporary circulating enterovirus D68 strains infect and undergo retrograde axonal transport in spinal motor neurons independent of sialic acid. J Virol 93:16. doi:10.1128/JVI.00578-19 PMC6675884 31167912

[11] Evans WJ, Hurst BL, Peterson CJ, Van Wettere AJ, Day CW, Smee DF, Tarbet EB. 2019. Development of a respiratory disease model for enterovirus D68 in 4-week-old mice for evaluation of antiviral therapies. Antiviral Res 162:61–70. doi:10.1016/j.antiviral.2018.11.012 30521834 PMC6997929

[12] Zhang C, Zhang X, Dai W, Liu Q, Xiong P, Wang S, Geng L, Gong S, Huang Z. 2018. A mouse model of enterovirus D68 infection for assessment of the efficacy of inactivated vaccine. Viruses 10:58. doi:10.3390/v10020058 29385753 PMC5850365

[13] Zheng H-W, Sun M, Guo L, Wang J-J, Song J, Li J-Q, Li H-Z, Ning R-T, Yang Z-N, Fan H-T, He Z-L, Liu L-D. 2017. Nasal infection of enterovirus D68 leading to lower respiratory tract pathogenesis in ferrets (Mustela putorius furo). Viruses 9:104. doi:10.3390/v9050104 28489053 PMC5454417

[14] Esposito S, Chidini G, Cinnante C, Napolitano L, Giannini A, Terranova L, Niesters H, Principi N, Calderini E. 2017. Acute flaccid myelitis associated with enterovirus-D68 infection in an otherwise healthy child. Virol J 14:4. doi:10.1186/s12985-016-0678-0 28081720 PMC5234096

[15] Kusabe Y, Takeshima A, Seino A, Nishida M, Takahashi M, Yamada S, Shimbo J, Sato A, Okamoto K, Igarashi S. 2017. [An adult case of enterovirus D68 encephalomyelitis presenting as bilateral facial nerve palsy and dysphagia]. Brain Nerve 69:957–961. doi:10.11477/mf.1416200848 28819079

[16] Chong PF, Kira R, Mori H, Okumura A, Torisu H, Yasumoto S, Shimizu H, Fujimoto T, Hanaoka N, Kusunoki S, Takahashi T, Oishi K, Tanaka-Taya K, Acute Flaccid Myelitis Collaborative Study Investigators. 2018. Clinical features of acute flaccid myelitis temporally associated with an enterovirus D68 outbreak: results of a nationwide survey of acute flaccid paralysis in Japan, August-December 2015. Clin Infect Dis 66:653–664. doi:10.1093/cid/cix860 29028962 PMC5850449

[17] Imamura T, Suzuki A, Lupisan S, Kamigaki T, Okamoto M, Roy CN, Olveda R, Oshitani H. 2014. Detection of enterovirus 68 in serum from pediatric patients with pneumonia and their clinical outcomes. Influenza Other Respir Viruses 8:21–24. doi:10.1111/irv.12206 24209770 PMC4177794

[18] ElBadry M, Lednicky J, Cella E, Telisma T, Chavannes S, Loeb J, Ciccozzi M, Okech B, Beau De Rochars VM, Salemi M, Morris Jr JG. 2016. Isolation of an enterovirus D68 from blood from a child with pneumonia in rural Haiti: close phylogenetic linkage with New York strain. Pediatr Infect Dis J 35:1048–1050. doi:10.1097/INF.0000000000001283 27331858

[19] Van Haren K, Ayscue P, Waubant E, Clayton A, Sheriff H, Yagi S, Glenn-Finer R, Padilla T, Strober JB, Aldrovandi G, Wadford DA, Chiu CY, Xia D, Harriman K, Watt JP, Glaser CA. 2015. Acute flaccid myelitis of unknown etiology in California, 2012-2015. JAMA 314:2663–2671. doi:10.1001/jama.2015.17275 26720027

[20] Kreuter JD, Barnes A, McCarthy JE, Schwartzman JD, Oberste MS, Rhodes CH, Modlin JF, Wright PF. 2011. A fatal central nervous system enterovirus 68 infection. Arch Pathol Lab Med 135:793–796. doi:10.5858/2010-0174-CR.1 21631275

[21] Mena I, Perry CM, Harkins S, Rodriguez F, Gebhard J, Whitton JL. 1999. The role of B lymphocytes in coxsackievirus B3 infection. Am J Pathol 155:1205–1215. doi:10.1016/S0002-9440(10)65223-6 10514403 PMC1867001

[22] Wahid R, Cannon MJ, Chow M. 2005. Dendritic cells and macrophages are productively infected by poliovirus. J Virol 79:401–409. doi:10.1128/JVI.79.1.401-409.2005 15596833 PMC538697

[23] Shen L, Chen CY, Huang D, Wang R, Zhang M, Qian L, Zhu Y, Zhang AZ, Yang E, Qaqish A, Chumakov K, Kouiavskaia D, Vignuzzi M, Nathanson N, Macadam AJ, Andino R, Kew O, Xu J, Chen ZW. 2017. Pathogenic events in a nonhuman primate model of oral poliovirus infection leading to paralytic poliomyelitis. J Virol 91:14. doi:10.1128/JVI.02310-16 PMC5487571 28356537

[24] Kramer M, Schulte BM, Toonen LWJ, de Bruijni MAM, Galama JMD, Adema GJ, van Kuppeveld FJM. 2007. Echovirus infection causes rapid loss-of-function and cell death in human dendritic cells. Cell Microbiol 9:1507–1518. doi:10.1111/j.1462-5822.2007.00888.x 17298395

[25] Lin YW, Wang SW, Tung YY, Chen SH. 2009. Enterovirus 71 infection of human dendritic cells. Exp Biol Med (Maywood) 234:1166–1173. doi:10.3181/0903-RM-116 19596831

[26] Racaniello VR. 2006. One hundred years of poliovirus pathogenesis. Virology 344:9–16. doi:10.1016/j.virol.2005.09.015 16364730

[27] Solomon T, Lewthwaite P, Perera D, Cardosa MJ, McMinn P, Ooi MH. 2010. Virology, epidemiology, pathogenesis, and control of enterovirus 71. Lancet Infect Dis 10:778–790. doi:10.1016/S1473-3099(10)70194-8 20961813

[28] Zhao T, Zhang Z, Zhang Y, Feng M, Fan S, Wang L, Liu L, Wang X, Wang Q, Zhang X, Wang J, Liao Y, He Z, Lu S, Yang H, Li Q. 2017. Dynamic interaction of enterovirus 71 and dendritic cells in infected neonatal rhesus macaques. Front Cell Infect Microbiol 7:171. doi:10.3389/fcimb.2017.00171 28540257 PMC5423916

[29] Xing J, Wang K, Wang G, Li N, Zhang Y. 2022. Recent advances in enterovirus A71 pathogenesis: a focus on fatal human enterovirus A71 infection. Arch Virol 167:2483–2501. doi:10.1007/s00705-022-05606-4 36171507

[30] He Y, Ong KC, Gao Z, Zhao X, Anderson VM, McNutt MA, Wong KT, Lu M. 2014. Tonsillar crypt epithelium is an important extra-central nervous system site for viral replication in EV71 encephalomyelitis. Am J Pathol 184:714–720. doi:10.1016/j.ajpath.2013.11.009 24378407

[31] Smura T, Ylipaasto P, Klemola P, Kaijalainen S, Kyllönen L, Sordi V, Piemonti L, Roivainen M. 2010. Cellular tropism of human enterovirus D species serotypes EV-94, EV-70, and EV-68 in vitro: implications for pathogenesis. J Med Virol 82:1940–1949. doi:10.1002/jmv.21894 20872722

[32] Laksono BM, Grosserichter-Wagener C, de Vries RD, Langeveld SAG, Brem MD, van Dongen JJM, Katsikis PD, Koopmans MPG, van Zelm MC, de Swart RL. 2018. In vitro measles virus infection of human lymphocyte subsets demonstrates high susceptibility and permissiveness of both naive and memory B cells. J Virol 92:e00131-18. doi:10.1128/JVI.00131-18 29437964 PMC5874404

[33] Sakabe S, Iwatsuki-Horimoto K, Takano R, Nidom CA, Le MTQ, Nagamura-Inoue T, Horimoto T, Yamashita N, Kawaoka Y. 2011. Cytokine production by primary human macrophages infected with highly pathogenic H5N1 or pandemic H1N1 2009 influenza viruses. J Gen Virol 92:1428–1434. doi:10.1099/vir.0.030346-0 21367984 PMC3168279

[34] Videira PA, Amado IF, Crespo HJ, Algueró MC, Dall’Olio F, Cabral MG, Trindade H. 2008. Surface alpha 2-3- and alpha 2-6-sialylation of human monocytes and derived dendritic cells and its influence on endocytosis. Glycoconj J 25:259–268. doi:10.1007/s10719-007-9092-6 18080182

[35] Eash S, Tavares R, Stopa EG, Robbins SH, Brossay L, Atwood WJ. 2004. Differential distribution of the JC virus receptor-type sialic acid in normal human tissues. Am J Pathol 164:419–428. doi:10.1016/S0002-9440(10)63132-X 14742248 PMC1602281

[36] Cao W, Liu YJ. 2007. Innate immune functions of plasmacytoid dendritic cells. Curr Opin Immunol 19:24–30. doi:10.1016/j.coi.2006.11.004 17113765

[37] Sioofy-Khojine A-B, Richardson SJ, Locke JM, Oikarinen S, Nurminen N, Laine A-P, Downes K, Lempainen J, Todd JA, Veijola R, Ilonen J, Knip M, Morgan NG, Hyöty H, Peakman M, Eichmann M. 2022. Detection of enterovirus RNA in peripheral blood mononuclear cells correlates with the presence of the predisposing allele of the type 1 diabetes risk gene IFIH1 and with disease stage. Diabetologia 65:1701–1709. doi:10.1007/s00125-022-05753-y 35867130 PMC9477938

[38] Kwakkenbos MJ, Diehl SA, Yasuda E, Bakker AQ, van Geelen CMM, Lukens MV, van Bleek GM, Widjojoatmodjo MN, Bogers W, Mei H, Radbruch A, Scheeren FA, Spits H, Beaumont T. 2010. Generation of stable monoclonal antibody-producing B cell receptor-positive human memory B cells by genetic programming. Nat Med 16:123–128. doi:10.1038/nm.2071 20023635 PMC2861345

[39] Jenner J, Kerst G, Handgretinger R, Müller I. 2006. Increased alpha2,6-sialylation of surface proteins on tolerogenic, immature dendritic cells and regulatory T cells. Exp Hematol 34:1212–1218. doi:10.1016/j.exphem.2006.04.016 16939814

[40] Vuorinen T, Vainionpää R, Kettinen H, Hyypiä T. 1994. Coxsackievirus B3 infection in human leukocytes and lymphoid cell lines. Blood 84:823–829. doi:10.1182/blood.V84.3.823.823 8043865

[41] Horstmann DM. 1952. Poliomyelitis virus in blood of orally infected monkeys and chimpanzees. Proc Soc Exp Biol Med 79:417–419. doi:10.3181/00379727-79-19398 14920447

[42] Pham NTK, Thongprachum A, Baba T, Okitsu S, Trinh QD, Komine-Aizawa S, Shimizu H, Hayakawa S, Ushijima H. 2017. A 3-month-old child with acute gastroenteritis with enterovirus D68 detected from stool specimen. Clin Lab 63:1269–1272. doi:10.7754/Clin.Lab.2017.170219 28792716

[43] Antona D, Kossorotoff M, Schuffenecker I, Mirand A, Leruez-Ville M, Bassi C, Aubart M, Moulin F, Lévy-Bruhl D, Henquell C, Lina B, Desguerre I. 2016. Severe paediatric conditions linked with EV-A71 and EV-D68, France, May to October 2016. Euro Surveill 21:11–14. doi:10.2807/1560-7917.ES.2016.21.46.30402 PMC5144948 27918268

[44] Chang TH, Yang TI, Hsu WY, Huang LM, Chang LY, Lu CY. 2019. Case report: painful exanthems caused by enterovirus D68 in an adolescent. Medicine (Baltimore) 98:e16493. doi:10.1097/MD.0000000000016493 31415349 PMC6831410

[45] Zhu Q, Berzofsky JA. 2013. Oral vaccines: directed safe passage to the front line of defense. Gut Microbes 4:246–252. doi:10.4161/gmic.24197 23493163 PMC3669171

[46] van Binnendijk RS, Poelen MC, de Vries P, Voorma HO, Osterhaus AD, Uytdehaag FG. 1989. Measles virus-specific human T cell clones. Characterization of specificity and function of CD4+ helper/cytotoxic and CD8+ cytotoxic T cell clones. J Immunol 142:2847–2854. 2467943

[47] Wang Y, Wang F, Wang R, Zhao P, Xia Q. 2015. 2A self-cleaving peptide-based multi-gene expression system in the silkworm Bombyx mori. Sci Rep 5:16273. doi:10.1038/srep16273 26537835 PMC4633692

[48] Atkinson GF. 1961. The Spearman-Karber method of estimating 50% endpoints. Contract No.: BU-141-M. Dept. of Biometrics: Biometrics Unit Technical Reports. Cornell University

