# Supplementary figures and images for: Human B cells and dendritic cells are susceptible and permissive to enterovirus D68 infection
Source: mSphere. 2024 Jan 23;9(2):e00526-23. doi: 10.1128/msphere.00526-23 (PMC10900886; doi:10.1128/msphere.00526-23)

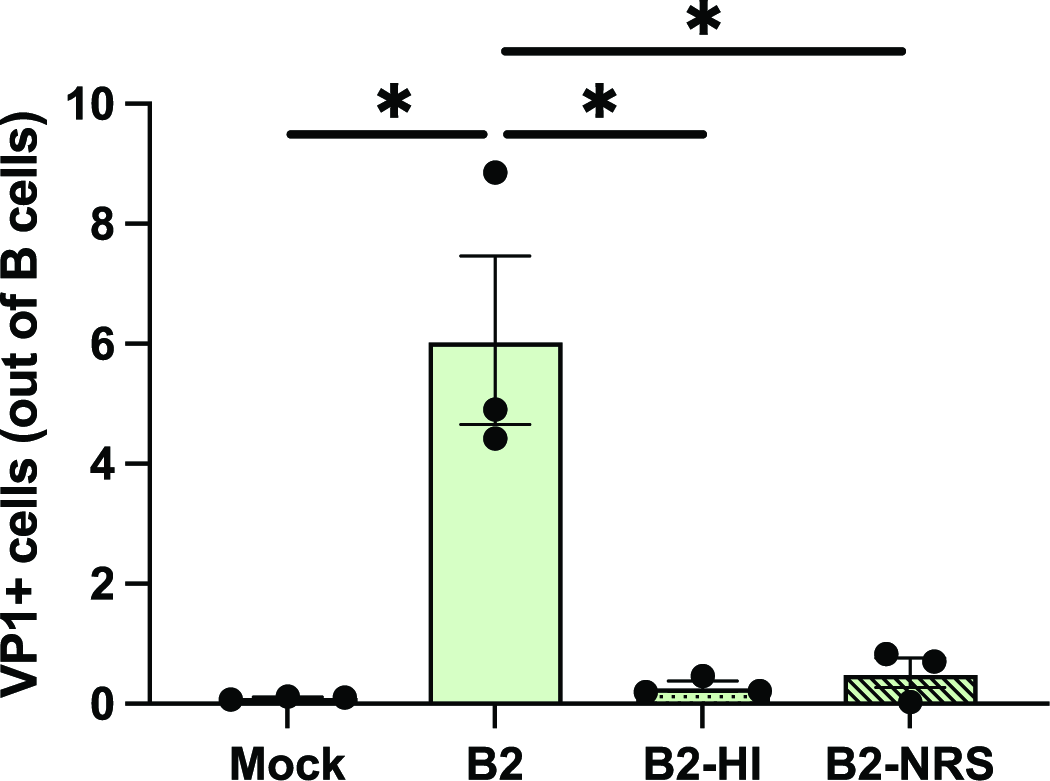

Supplement: Fig. S1 — PBMC VP1 staining controls. [file msphere.00526-23-s0001.tif]

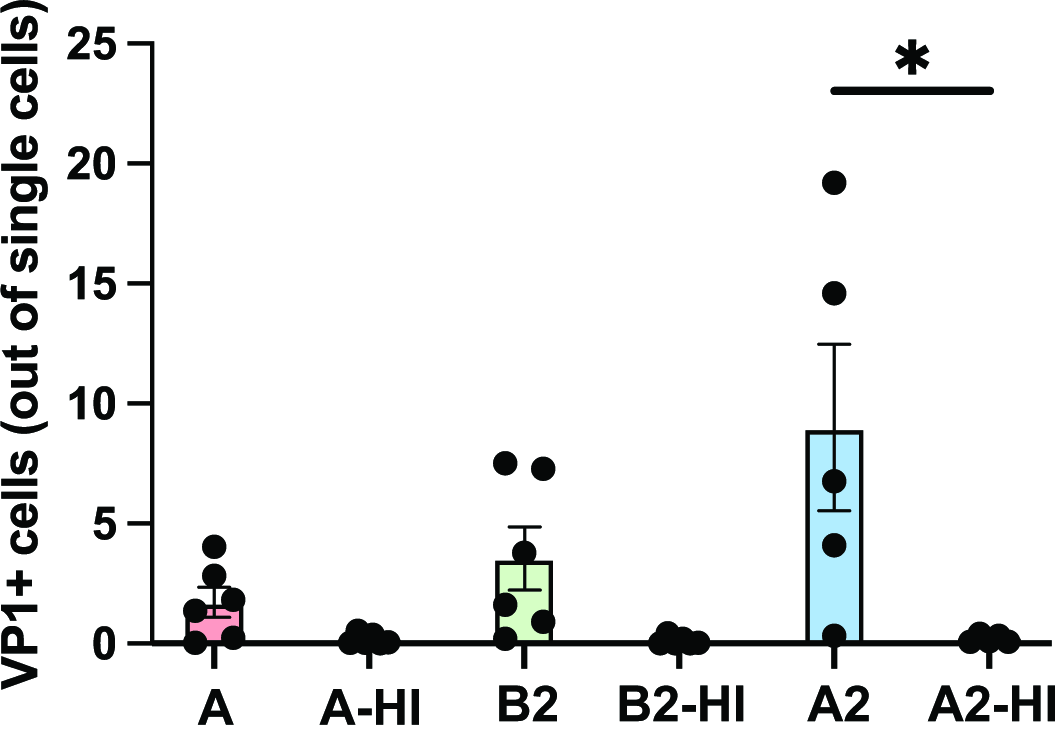

Supplement: Fig. S2 — BLCL heat-inactivated controls. [file msphere.00526-23-s0002.tif]

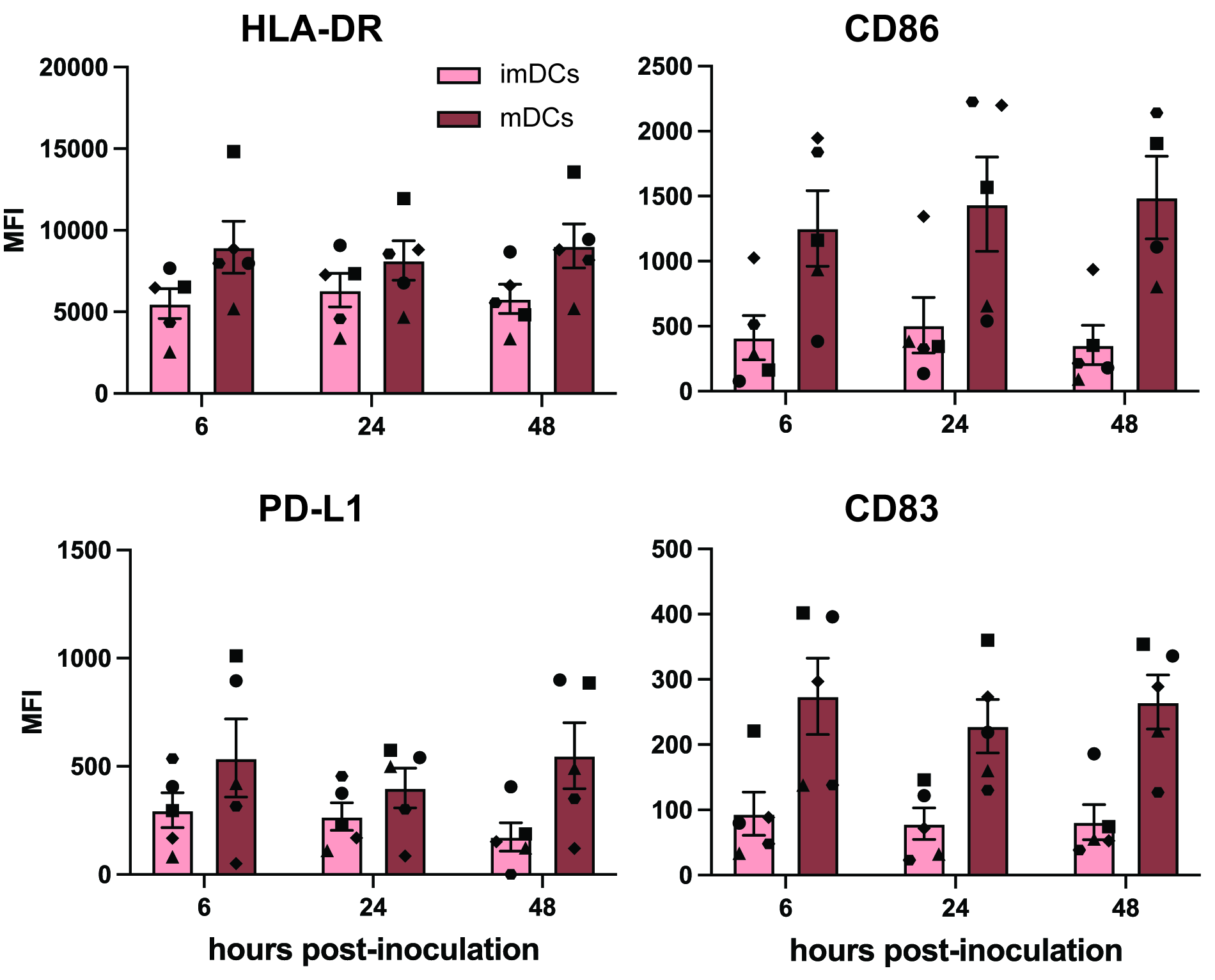

Supplement: Fig. S3 — Maturation markers of immature and mature DCs. [file msphere.00526-23-s0003.tif]
