# Supplementary material for: Human B cells and dendritic cells are susceptible and permissive to enterovirus D68 infection
Source: mSphere. 2024 Jan 23;9(2):e00526-23. doi: 10.1128/msphere.00526-23 (PMC10900886; doi:10.1128/msphere.00526-23)
Supplement: Supplemental Material Legends — Legends for Fig. S1-S3. [file msphere.00526-23-s0004.docx]

**Supplementary Information**

**S1. Heat-inactivated virus and isotype controls for the inoculation of PBMC with EV-D68/B2.** Inoculation of PBMCs with EV-D68/B2, but not with heat-inactivated EV-D68/B2 (B2-HI), results in detection of VP1^+^ signal in B cells. EV-D68/B2-inoculated PBMC were incubated with normal rabbit serum (B2-NRS) as a staining isotype control. Statistical analysis was performed with unpaired t-test. *: P<0.05

**S2. Heat-inactivated virus controls for the inoculation of BLCL with EV-D68.** BLCL were inoculated with EV-D68/A (n = 4), EV-D68/B2 (n = 4), EV-D68/A2 (n = 5) and their heat-inactivated counterparts (A-HI, B2-HI and A2-HI). VP1^+^ cells were only detected in cells inoculated with virus, and not when BLCLs were inoculated with heat-inactivated virus. Statistical analysis was performed with unpaired t-test. *: P<0.05

**S3.** **The expression of dendritic cell maturation markers in immature and mature dendritic cells (imDCs and mDCs).** mDCs were defined by the upregulation of surface HLA-DR, CD86, PD-L1 and CD83 after treatment of monocyte-derived imDCs with lipopolysacharride. Each symbol represents one donor. Error bars denote standard error of mean. MFI: median fluorescence intensity.
